# Status of Planned and Ongoing Paediatric Trials Investigating COVID-19 Vaccines: A Cross-Sectional Study of Paediatric Clinical Trials Planned in Agreed PIPs and/or Registered in Clinical Trial Databases

**DOI:** 10.1007/s43441-021-00356-y

**Published:** 2022-02-07

**Authors:** Helle Christiansen, Steffen Thirstrup, Christine Erikstrup Hallgreen

**Affiliations:** 1 Department of Pharmacy, Copenhagen Centre for Regulatory Science, University of Copenhagen, Universitetsparken 2, 2100 Copenhagen, Denmark; 2 NDA Group AB, Johanneslundsvägan 2, 194 91, Upplands Väsby, Sweden

**Keywords:** Paediatric, COVID-19 vaccines, Drug development, Paediatric clinical trials

## Abstract

**Background:**

The immune system matures throughout childhood; therefore, evidence about the safety and efficacy of vaccines for the prevention of COVID-19 in the paediatric population is important. Efficacy and safety have not been established for COVID-19 vaccines in a large part of the paediatric population at the time of the initial approval for use in adults. This study aims to provide an overview of planned and ongoing paediatric clinical trials investigating the safety and/or efficacy of COVID-19.

**Methods:**

We identified all paediatric clinical trials investigating the safety and/or efficacy of COVID-19 vaccines in clinicaltrials.gov and clinicaltrialregister.eu, as well as all clinical trials planned in agreed PIPs (Paediatric Investigational Plans) as of 11 June 2021. Information about the study design, the paediatric age groups that they included, and the primary and secondary safety and efficacy outcomes were collected, together with expected timelines for the studies.

**Results:**

21 clinical trials were identified through the clinical trial registries and 19 clinical trials were specified in 6 agreed PIPs, 5 of these trials were also in the trial registers. All PIPs stipulated development of the COVID-19 vaccines for the full paediatric population, with a deferral. The earliest expected completion date of a PIPs is March 2024. The majority (14/21) of registered trials are randomised double-blinded studies. All investigated safety, 20 have a surrogate efficacy outcome (immunogenicity), of these 7 also measure clinical efficacy (COVID-19 infections). 18 studies were initiated, of these, all but one is still ongoing and one in adolescents has been finalised.

**Conclusion:**

Even though several trials have been planned in agreed PIPs, the registered paediatric clinical trials identified are most often not part of a PIP.

**Supplementary Information:**

The online version contains supplementary material available at 10.1007/s43441-021-00356-y.

## Introduction

The rapid development and implementation of vaccines against COVID-19 are society’s best weapon against the social, economic and human devastation brought about by the COVID-19 pandemic. Along with the approval of COVID-19 vaccines for the adult population, many have highlighted the urgency of an ethical and effective strategy to implement the COVID-19 vaccines for the paediatric population as rapidly and safely as possible [1, 2]. The paediatric population is defined as the ‘part of the population aged between birth and 18 years’ in the European Paediatric Regulation (EPR) [3]. However, safety and efficacy in a large proportion of the paediatric population have not been established for COVID-19 vaccines at the time of initial approval for use in adults [4].

In the EU (European Union), four COVID-19 vaccines have currently been granted a CMA (Conditional Marketing Approval) [5–8]. They are manufactured by Pfizer-BioNTech, Moderna, AstraZeneca and Janssen. Three of these four vaccines were only approved for use in adults [5–7]. The Pfizer-BioNTech vaccine was, however, initially approved for use in adults and adolescents aged 16 or older [6]. This indication was extended on 28 May 2021 to include adolescents aged 12 years and above [9]. The EMA (European Medicines Agency) is currently also evaluating the Moderna COVID-19 vaccine for use in adolescents (12 to 17 years of age) [10].

Since the EPR [3] came into effect in 2007, it has been mandatory for Marketing Authorisation Applicants, to submit the results from studies conducted as part of an agreed PIP (Paediatric Investigational Plan) when applying for a MA (Marketing Authorisation), unless granted a waiver or a deferral. The regulation was set in place to stimulate the research of medicines for use in the paediatric population and to provide more information about the use of medicines in this population. The agreed PIP describes the studies to be conducted in all relevant subsets of the paediatric population, including the timing of completion. However, the agreed PIP trials need not to be conducted as separate studies [3] and including adolescents in adult phase III clinical trials can be feasible when the disease and the response to a drug are likely to be similar [11]. Since the immune system develops and matures throughout childhood [12], establishing safety and efficacy is especially important if a vaccine is to be used in the paediatric population [1, 13, 14].

This study aims to provide insight into the timely drug development of COVID-19 vaccines for the paediatric population by investigating registered clinical trials on the safety and the efficacy of COVID-19 vaccines along with the planned clinical trials agreed in a PIP and the expected timelines for completion.

## Method

All paediatric clinical trials investigating a COVID-19 vaccine registered at clinicaltrials.gov or clinicaltrialregister.eu were identified as of 11 June 2021. A paediatric trial was defined as any clinical trial including subjects from any of the paediatric subgroups (adolescent, children, toddler and infants, and term newborn) as defined by the International Conference on Harmonization (ICH) Topic E 11, 2001 [15]. Clinicaltrial.gov was searched with the terms “COVID-19”, “Corona virus infection”, “SARS-CoV-2” and “Severe Acute Respiratory Syndrome” filtering for ‘Child (birth-17)’ as eligibility criteria and ‘Interventional (Clinical trial)’ as study type. Clinicaltrialregister.eu was searched with the terms “COVID-19”, “Corona virus infection”, “SARS-CoV-2” and “Severe Acute Respiratory Syndrome” combined with selected age range set to ‘under 18 years’. We collected trial design, clinical trial phases, age of participants (separated into the paediatric subgroups as defined by the International Conference on Harmonization (ICH) Topic E 11, 2001 [15]: adolescents 12–18 years, children 2–11 years, infants and toddlers 28 days to 23 months, and term newborn 0–27 days), primary and secondary outcome measures (safety, surrogate efficacy outcome (immunogenicity) and/or clinical efficacy outcome (confirmed COVID-19 infection)), together with longest primary or secondary safety follow-up, study start date and completion date.

Then, we identified all agreed PIPs with an active substance investigated for the prevention of COVID-19 in the EU as of 17 June 2021, through the website of the European Medicines Agency’s (EMA) [16]. The Paediatric Committee (PDCO) decision (waiver and/or agreed PIP and deferral hereof), the date of expected completion of the PIP and information on planned studies were collected. The EU approval status and approval date for each active substance targeted by a PIP were retrieved by matching the PDCO decision number from the European Public Assessment Reports (EPARs) of an approved COVID-19 vaccine at the time of the MA with those of the PIP. The EPARs were also retrieved from the EMA website.

For clinical trials registered in the clinicaltrialregister.eu, we recorded if the sponsor reported the trial to be part of a PIP (section A.7. in the EudraCT form). For clinical trials registered in the Clinicaltrial.gov, we evaluated if a current trial was likely to be part of a PIP by matching the trial IDs provided, trial design, outcome measures and the age groups involved.

## Results

**Fig. 1:**
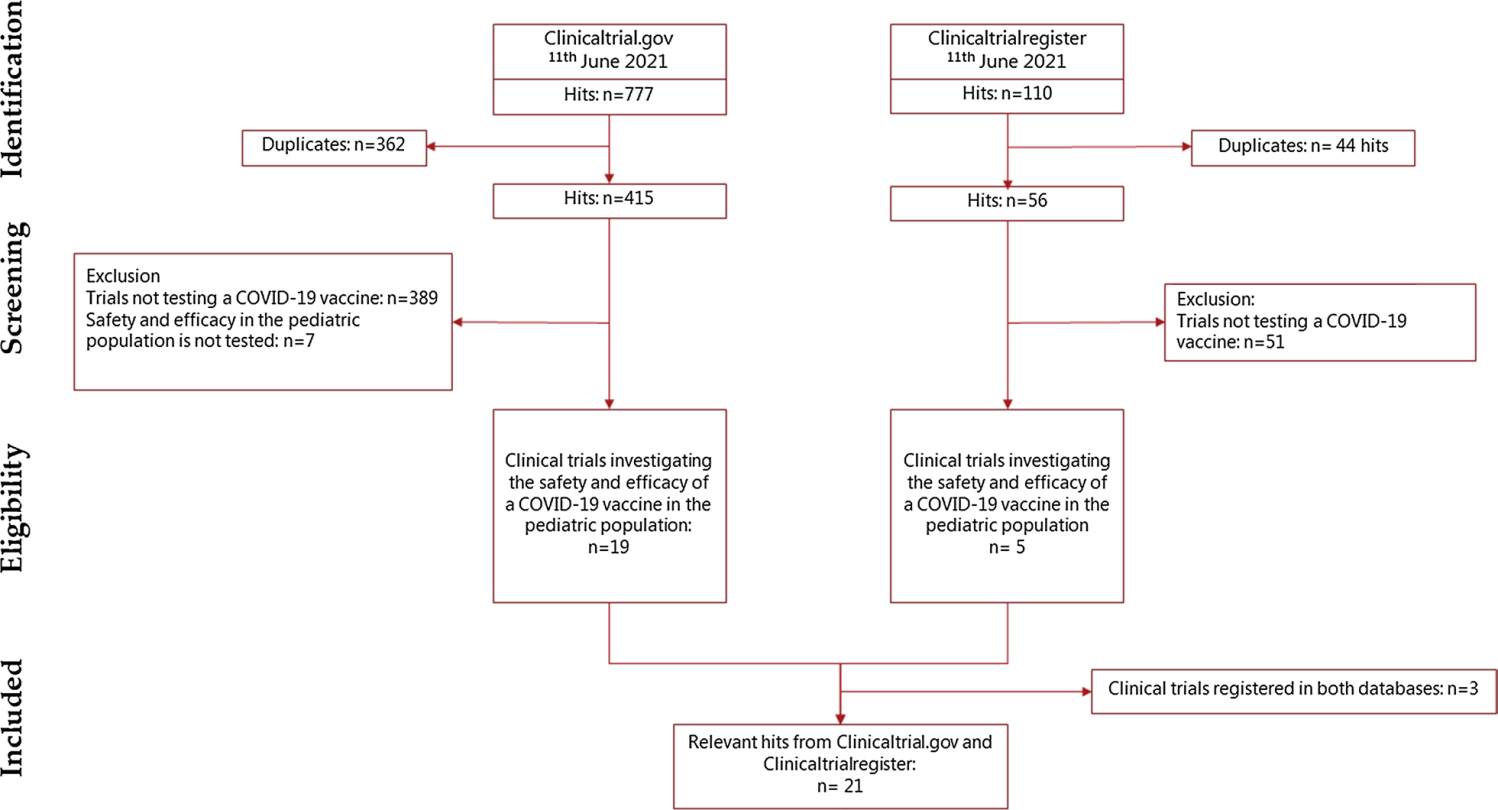
Flowchart of the study population selection

**Fig. 2:**
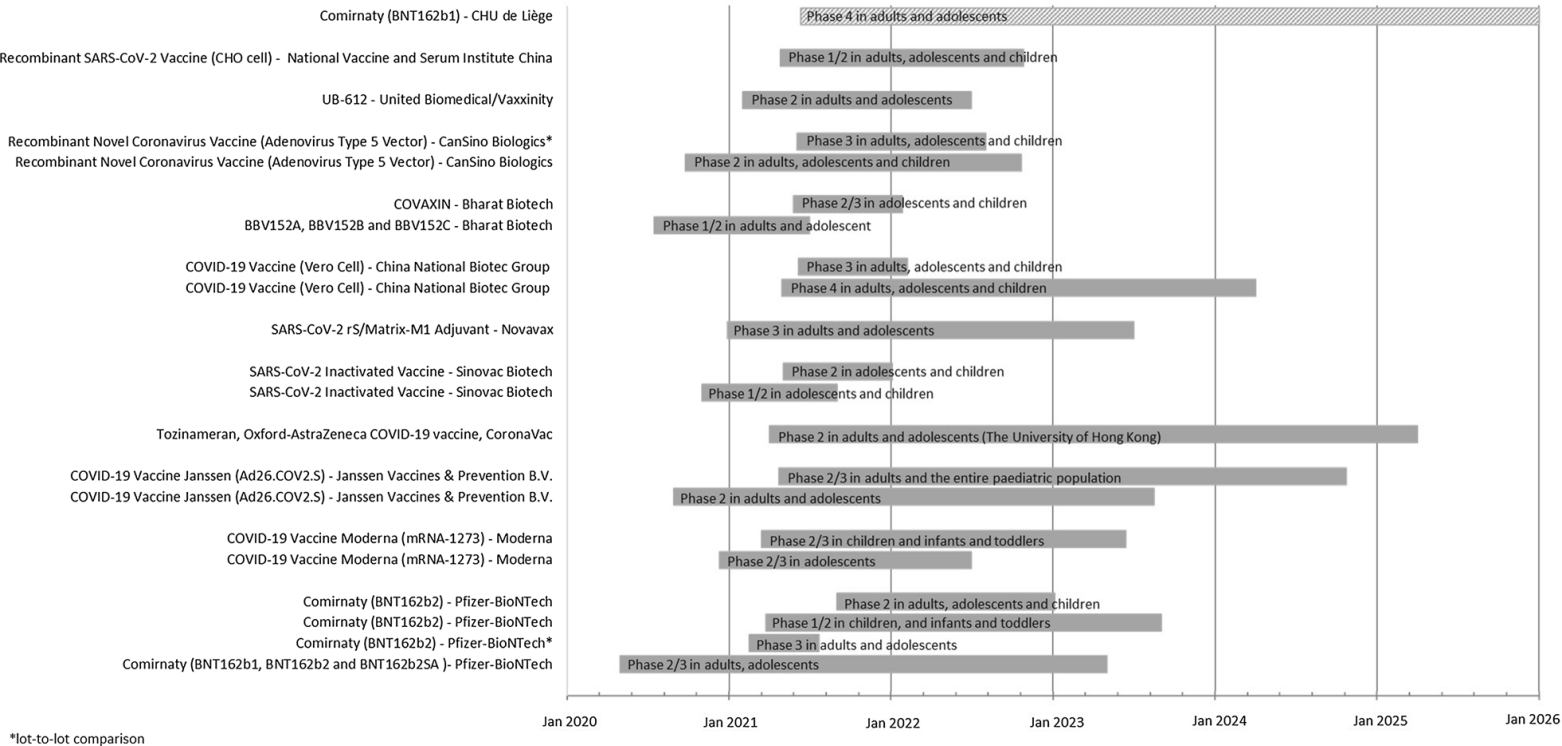
Overview of the start and completion dates for all registered paediatric clinical trials in clinicaltrial.gov and/or clinicaltrialregister.eu (*N* = 21) Grey bars represent study timeline, the study is represented by the shaded bar in the top has not reported expected study start or end, the bar starts on the date of first reported in EudraCT. EU vaccination dates, Comirnaty 21/12 2020, COVID-19 vaccine Moderna 06/01 2021, Vaxzevira 29/01 2021 and COVID-19 vaccine Janssen 11/03 2021

A total of 21 clinical trials testing the safety and efficacy of a COVID-19 vaccine in the paediatric population were identified in clinicaltrial.gov and/or clinicaltrialregister.eu (Fig. 1). Of these, 10 clinical trials investigated a vaccine approved in the EU (see Fig. 2). No AstraZeneca-sponsored studies in the paediatric population was identified. In July 2021, the first trial is expected to be completed for adolescents. This is a Phase III study by Pfizer-BioNTech evaluating the safety and immunogenicity of Comirnaty (BNT162b2) against COVID-19 in healthy participants, which includes adolescents, comparing manufacturing lots, as well as a booster vaccine. Sionvac Biotech are the first expecting to finalise clinical trials including children; a Phase 1/2 in September 2021 and a Phase 2 in January 2022. The first trial including infants and toddlers is a Phase II/III investigating the safety of Moderna COVID-19 vaccine which will be completed in June 2023 (see Fig. 2). For term newborn, one clinical trial was registered to investigate the safety and efficacy of a COVID-19 vaccine (Janssen COVID‑19 vaccine). This trial is expected to be completed in October 2024. Most (15/21) registered clinical trials were randomised control trials, all but one was registered as double blinded (14/21). All but one of the registered studies will be investigating safety and efficacy, where efficacy is measured by the surrogate endpoint of immunogenicity (see Table 1). In addition, seven of the studies will investigate clinical efficacy as the incidence of COVID-19 infection after vaccination, two where the COVID-19 infections are self-reported by study participants (data not shown).

**Table 1:** Characteristics of registered clinical trials investigating safety and/or efficacy of COVID-19 vaccines in the paediatric population as of 11 June 2021

	No. (%)			Comparator		Primary or secondary end point			Months (IQR) Median safety follow-up
	Randomised	Open label	Double blind	Active	Placebo	Safety	Surrogate outcome	Clinical outcome^g^	
All (*N* = 21)^d,e^	15^f^ (71)	7 (33)^h^	14 (67)	1 (5)	12 (57)	21 (100)	20 (95)	7 (33)	12 (6–12)
Paediatric development stage									
Exploratory^a^ (*N* = 10)	7	3	7	1	7	10	9	2	12 (6–21)
Confirmatory^b^ (*N* = 11)	8^e^	4	7	0	5	11	11	5	12 (6–12)
Age^i^									
Adolescents (*12–18 years*^c^) (*N* = 19)	14	6	13	1	11	19	18	5	10.5 (6–12)
Children (2–11 years) (*N* = 12)	8	5	7	0	6	12	11	2	12 (6–12)
Toddlers and infants (28 days–2 years) (*N* = 3)	2	1	2	0	2	3	3	2	12 (12—18)
Term newborn (0–27 days) (*N* = 1)	1	0	1	0	1	1	1	0	12 (-)

^a^Defined as phase I, Phase I/II or Phase II clinical trials

^b^Defined as phase II/III or phase III clinical trials

^c^In the US, 12–17 years

^d^10 trials on vaccines approve in EU,

^e^Five trials part of an agreed PIP,

^f^Two study compared multiple lots

^g^Clinical outcome measure was always in combination with surrogate outcome measure of immunogenicity

^h^Two studies in immunosuppressed

^i^The studies can include several paediatric subsets as well as adults

In the EU, six PIPs for a COVID-19 vaccine had been agreed, all covering a development for the entire paediatric population. Four of these PIPs are related to currently EU-approved vaccines (see Table 1). One of the six PIPs was related to the paediatric development of a vaccine currently under rolling review by EMA (SARS-CoV-2 rS from Novavax, Inc.), and one was related to a vaccine (CoV-2 preS dTM from Sanofi Pasteur) currently not under evaluation by EMA. All the PIPs have been deferred until after approval in the adult population, with completion dates between March 2024 and March 2025 (see Table 2).

**Table 2:** Characteristics of clinical trials in agreed Paediatric Investigational Plans (PIP) as of 11 June 2021

	No. of agreed clinical trials					Comparator		Primary or Secondary End Point			Population	
	Medicines names	Randomised	Open label	Double blind	Observer blind	Active	Placebo	Safety	Surrogate outcome	Clinical outcome	Healthy	Immunesup
All (*N* = 19)	–	14	6	5	8	3	12	19	19	2	13	6
PIPs with approved vaccines												
BNT162b2 (*N* = 4)	Comirnaty	3	2	2	0	2	2	4	4	1	3	1
mRNA-1273 (*N* = 3)	COVID-19 Vaccine Moderna	2	1	0	2	0	2	3	3	0	2	1
Ad26.COV2-S (*N* = 3)	COVID-19 Vaccine Janssen	2	1	2	0	0	2	3	3	0	2	1
ChAdOx1-S (*N* = 3)	Vaxzevria	2	1	2	0	0	2	3	3	0	2	1
PIPs with unapproved vaccines												
SARS-CoV-2 rS (*N* = 4)	NVX-CoV2373	4	0	0	4	1	3	4	4	1	3	1
CoV-2 preS dTM (*N* = 2)	–	1	1	1	0	1	1	2	2	0	1	1
Paediatric age groups included (all PIPs)												
Adolescents (12–18 yearsc) (*N* = 15)	–	10	5	4	6	2	9	15	15	2	9	6
Children (2–11 years) (*N* = 14)	–	9	5	3	6	1	9	14	14	1	8	6
Toddlers and infants (28 days–2 years) (*N* = 14)	–	9	6	3	5	2	8	14	14	0	8	6
Term newborn (0–27 days) (*N* = 13)	–	8	6	2	5	2	7	13	13	0	7	6

In total, 19 clinical studies were planned for the paediatric population as part of an agreed PIP. Each PIP contained 2–4 planned clinical trials and had at least one trial in immunosuppressed paediatric patients. Five trials were already registered in clinicaltrial.gov and/or clinicaltrialregister.eu (Table 1). Fifteen of the PIP-agreed clinical trials included adolescents, 14 trials included children, and toddlers and infants, and 13 trials included term newborn (see Table 2). Safety and efficacy (specified as immunogenicity) are to be investigated in all PIP-agreed clinical studies, two are also to measure clinical efficacy as incidence of COVID-19 infection after vaccination (see Table 2). PIPs did not stipulate the need for quality-related studies, non-clinical studies, extrapolation modelling and simulation studies or other studies and measures.

## Discussion

Our study shows that clinical trials to investigate the safety and efficacy of all currently EU-approved COVID-19 vaccines had been registered in one of the clinical trial databases or planned in agreed PIP for the entire paediatric population (term newborn to adolescents). However, most often the registered trials included adolescents and children. A discrepancy was observed between planned clinical trials in agreed PIPs and registered clinical trials for infants and toddlers, and term newborn. Only one study was registered for term newborn even though thirteen were planned in the agreed PIPs for COVID-19 vaccines. Similarly, there was a discrepancy observed between planned clinical trials with immunosuppressed paediatric patients in agreed PIPs and registered paediatric studies in this population. This may indicate that such trials although agreed with the authorities have not yet been fully planned and initiated.

In general, only a few of the trials registered were conducted as part of an agreed PIP, which indicated that the development is not driven by the EPR. However, if an agreed PIP contains requirements that has already been studied, this should not lead to duplication of research. The initial agreed PIPs have shown to be dynamic with many modifications introduced based on feasibility and changed in the development [17]. Also, even though studies in the PIPs only concern the paediatric population, this does not necessarily call for separate paediatric trials. Indeed, a majority of the identified COVID-19 vaccine trials including the paediatric population or subsets of the paediatric population were not separate paediatric trials, but also include the adult population.

Also, the paediatric COVID-19 vaccine development has progressed very fast with only five months delay from approval in individuals above 16 years of age to the extension of the indication to cover individuals 12 years of age and older as well [18]. For innovative therapeutics, the median time between regulatory approval in adults and the approval in the paediatric population is 5.6 years [19]. This could indicate that market forces are driving the COVID-19 vaccine development for adolescent and children, not the agreed PIPs. Four of the six PIP applications initially contained a waiver for conducting studies in subsets of the paediatric population. These were, however, later withdrawn assumingly because of PDCO’s evaluation. If this is in fact the case, the agreed PIPs could play a role in stimulating the development of the COVID-19 vaccines for the younger paediatric populations.

Chronological age will in many cases be an inappropriate proxy for physiological development and disease presentation relevant to medicines development in the paediatric population [20, 21]. The same most likely holds for vaccine development where maturation of the immune system [11] does not necessarily correlate with the paediatric subset as defined by regulatory authorities. In our study, we observe PIP-agreed studies for adolescents, however this population was in many cases included in the pivotal trials with adults. However, if sponsors do not generate the evidence needed for safe and effective use of their product in all relevant populations, the burden of medical uncertainty is laid upon the groups not represented in the drug development program [22]. The relevance of this with respect to COVID-19 vaccines is clearly exemplified by the recent observation that suggest a disproportionate higher frequency of post-vaccination myocarditis in adolescents and young adults than in the average adult population [23].

Four vaccines are currently (June 11, 2021) under rolling review by EMA (CVnCoV, NVX-CoV2373, Sputnik V, Vero Cell) [24]. Only one of these has an agreed PIP listed on the EMA website (NVX-CoV2373), even though COVID-19 Vaccine (Vero Cell) was registered to be investigating the safety and immunogenicity of adolescents and children in two confirmatory clinical trials.

As mentioned, the Pfizer-BioNTech Covid-19 vaccine was approved for adolescents 12 to 17 years of age on 28 May 2021. The study providing the basis for the paediatric indication was carried out in accordance with the PIP for the Pfizer-BioNTech COVID-19 vaccine and enrolled 2260 adolescents aged 12–15 [9]. However, this study has not yet reached the completed date according to information registered by the sponsor in both clinicaltrial.gov and clinicaltrialregister.eu, and the approval must have been granted based on data from an interim analysis. For the EU-approved COVID-19 vaccines, clinical studies in children, infants and toddlers have only just been initiated and are not expected to be completed until mid-2023. The Janssen study to be completed at the end of 2024 will also include term newborn. Some reports have been made that Pfizer-BioNTech and Moderna will apply for EUA for children (2–11 years of age) in fall 2021 [25].

We found that for both registered studies and planned studies agreed as part of the PIP, efficacy is mostly measured as immunogenicity. Only a few measured incidences of COVID-19 infection after vaccination. A similar pattern was observed by Kesselheim, et.al. 2020 showing that for vaccines approved by the FDA between 2006 and 2020, 65% of the pivotal studies supporting the approval have immunogenicity as primary end point. In the paediatric population, this was even higher (80%) [26]. Immunogenicity is a surrogate measurement allowing for a quicker assessment of efficacy. This is in particular useful when efficacy on clinical endpoints has been established in another population (e.g. adults) and allows for a faster development.

All registered clinical trials had a primary or secondary safety outcome, most often in a randomised control trial, with a median safety follow-up of 12 months after last dose. Kesselheim et al*.* found that pivotal studies supporting the approval of a vaccine by the FDA between 2006 and 2020, had a median follow-up of 18 months, and for the studies in the paediatric population, the duration was at least 12 months [26]. The vaccines currently approved in the EU for prevention of COVID-19 were granted a CMA (conditional marketing authorisation) which can be granted when the benefit-risk balance is positive, but the level of evidence-uncertainty is higher than what is required for a regular approval. The CMA was based on interim analysis of data from phase II/III studies with a medium follow-up of only around two months after last dose [4–7]. The recent approval of the Pfizer-BioNTech vaccine for use in adolescents 12 to 15 years of age was based on similar uncertainty around long-term safety [27]. The CMA can be used in cases where the medical product fulfils the unmet medical needs of patients with seriously debilitating or life-threatening diseases, or as in the case of COVID-19, in an emergency situation in response to a public health threat [28].

For the paediatric population, COVID-19 does not seem to be a seriously debilitating or life-threatening disease. Studies have suggested that paediatric individuals are at a lower risk of developing COVID-19 and that they have the disease more mildly than adults do [29–34]. The paediatric population may play a part in transmission and in achieving herd immunity [1, 2, 35], although studies suggest that the paediatric population only plays a minor role in community transmission [33, 34]. Since the vaccines are to be used by healthy individuals, safety is of utmost importance, leaving little room for trade-off on the benefit–risk balance. The benefit–risk balance may be even narrower when developing a vaccine for the paediatric population. Discussions about the use of COVID-19 vaccines approved through the CMA pathway are warranted for children (younger than 12 years of age), as is the evidence needed for a standard approval. Similar discussions have been opened in the US with respect to the use of EUA (Emergency Use Approval) versus standard approval at an Advisory Committee meeting in June 2021 [36].

The overview of clinical trials for vaccines for the prevention of COVID-19 in the paediatric population provided here only represents a snapshot in time. The development is moving at unprecedented speed, as is obvious from the recent expansion in the approved indication of the Pfizer-BioNTech vaccine to adolescents 12–15 years of age in May 2021 [18, 37, 38], as well as Moderna’s application to extend the indication of its COVID-19 vaccine for use in adolescents [9]. Also, this overview is limited to information available in the public domain, specifically studies reported in Clinialtrial.gov, the EU clinicaltrialregister.eu and in the publicly available PIPs. The latter only provides a few details on the planned studies agreed as part of a PIP, and a timeline for the completion of the full paediatric development programme. What is more, the division of the paediatric population into developmental subgroups is to some extent arbitrary [15]. The categorical determinant does not take physiological development into considerations but follows chronological age alone. Post-puberty adolescents’ immune systems may be fully matured, which appears to have been considered by some vaccine developers, as, e.g. the initial Comirnaty vaccine authorisation included adolescents 16 year of age or older. Finally, many registered clinical trials were started as phase I for which the protocol was amended into phase II and later phase III (quasi-adaptive). Finally, when interpreting the study timelines, it is important to remember that some phase II/III studies start with the adult population and only include the paediatric population at a later stage.

## Conclusion

This study shows that clinical trials to investigate the safety and efficacy of all currently approved COVID-19 vaccines had been registered in one of the clinical trials databases or planned in an agreed PIP for most of the paediatric population (neonates to adolescents); however, this development does not seem to be driven by the agreed PIPs.

## Supplementary Information

Below is the link to the electronic supplementary material.

Supplementary file1 (DOCX 20 kb)
