# Supplementary material for: Status of Planned and Ongoing Paediatric Trials Investigating COVID-19 Vaccines: A Cross-Sectional Study of Paediatric Clinical Trials Planned in Agreed PIPs and/or Registered in Clinical Trial Databases
Source: Ther Innov Regul Sci. 2022 Feb 7;56(3):474–82. doi: 10.1007/s43441-021-00356-y (PMC8821866; doi:10.1007/s43441-021-00356-y)
Supplement: Supplementary file 1 — Supplementary file1 (DOCX 20 kb) [file 43441_2021_356_MOESM1_ESM.docx]

Suplementing material - S1

| NCT.Number  /EudraCT | First posted | Last update posted | Title | Status | Phases | Randomised | Double.blinded | Controlled | Placebo | Active.comparator | Safety ourcome | Clinical efficacy outcome | Immunogenicity outecome | Popuulation | Approved in EU | Part of agreed PIP |
| --- | --- | --- | --- | --- | --- | --- | --- | --- | --- | --- | --- | --- | --- | --- | --- | --- |
| NCT04713553 | Jan 19, 2021 | June 11, 2021 | A Phase 3 Study to Evaluate the Safety, Tolerability, and Immunogenicity of Multiple Production Lots and Dose Levels of BNT162b2 RNA-Based COVID-19 Vaccines Against COVID-19 in Healthy Participants | Recruiting | Phase III | yes | yes | yes* | no | no | yes | no | yes | 12 Years to 50 Years  (Child, Adult) - Healthy | yes | no |
| NCT04471519 | July 15, 2020 | May 20, 2021 | Whole-Virion Inactivated SARS-CoV-2 Vaccine (BBV152) for COVID-19 in Healthy Volunteers | Active, not recruiting | Phase I /Phase II | yes | yes | yes | yes | no | yes | no | yes | 12 Years to 65 Years (Child, Adult, Older Adult) - Healthy | no | no |
| NCT04551547 | Sep 16, 2020 | Nov 9, 2020 | Safety and Immunogenicity Study of Inactivated Vaccine for Prevention of COVID-19 | Recruiting | Phase I /Phase II | yes | yes | yes | yes | no | yes | no | yes | 3 Years to 17 Years  (Child)  - Healthy | no | no |
| NCT04884685 | May 13, 2021 | June 4, 2021 | Safety of an Inactivated SARS-CoV-2 Vaccine (CoronaVac) in Children and Adolescents | Active, not recruiting | Phase II | yes | yes | yes | yes | no | yes | no | no | 3 Years to 17 Years  (Child)  - Healthy | no | no |
| NCT04649151 | Dec 2, 2020 | June 7, 2021 | A Study to Evaluate the Safety, Reactogenicity, and Effectiveness of mRNA-1273 Vaccine in Adolescents 12 to <18 Years Old to Prevent COVID-19 | Active, not recruiting | Phase II /Phase III | yes | yes | yes | yes | no | yes | yes | yes | 12 Years to 17 Years (Child)  - Healthy | yes | yes |
| NCT04773067 | Feb 26, 2021 | June 7, 2021 | A Study to Evaluate UB-612 COVID-19 Vaccine in Adolescent, Younger and Elderly Adult Volunteers | Recruiting | Phase II | yes | yes | yes | yes | no | yes | no | yes | 12 Years to 85 Years (Child, Adult, Older Adult) | no | no |
| NCT04566770 | Sep 28, 2020 | Nov 27, 2020 | A Clinical Trial of A COVID-19 Vaccine Named Recombinant Novel Coronavirus Vaccine (Adenovirus Type 5 Vector) | Recruiting | Phase II | yes | yes | yes | yes | no | yes | no | yes | 6 Years and older  (Child, Adult, Older Adult) -- Healthy | no | no |
| NCT04869592 | May 3, 2021 | May 7, 2021 | A Clinical Trial to Evaluate the Recombinant SARS-CoV-2 Vaccine (CHO Cell) for COVID-19 | Recruiting | Phase I /Phase II | yes | yes | yes | yes | no | yes | no | yes | 3 Years and older  (Child, Adult, Older Adult) - Healthy | no | no |
| NCT04368728/  2020-002641-42 | April 30, 2020 | June 1, 2021 | Study to Describe the Safety, Tolerability, Immunogenicity, and Efficacy of RNA Vaccine Candidates Against COVID-19 in Healthy Individuals | Recruiting | Phase II /Phase III | yes | yes | yes | yes | no | yes | yes | yes | 12 Years and older  (Child, Adult, Older Adult)  - Healthy | yes | no |
| NCT04796896 | March 15, 2021 | May 21, 2021 | A Study to Evaluate Safety and Effectiveness of mRNA-1273 Vaccine in Healthy Children Between 6 Months of Age and Less Than 12 Years of Age | Recruiting | Phase II /Phase III | yes | yes | yes | yes | no | yes | yes | yes | 6 Months to 11 Years (Child)  - Healthy | yes | yes |
| NCT04611802 | Nov 2, 2020 | May 6, 2021 | A Study to Evaluate the Efficacy, Immune Response, and Safety of a COVID-19 Vaccine in Adults â‰¥ 18 Years With a Pediatric Expansion in Adolescents (12-17 Years) at Risk for SARS-CoV-2 | Recruiting | Phase III | yes | yes | yes | yes | no | yes | yes | yes | 12 Years and older  (Child, Adult, Older Adult) - Healthy | no | no |
| NCT04535453/  2020-002584-63 | Sep 2, 2020 | April 20, 2021 | A Study to Evaluate a Range of Dose Levels and Vaccination Intervals of Ad26.COV2.S in Healthy Adults and Adolescents | Suspended (ongoing DE and NL) | Phase II | yes | yes | yes | yes | no | yes | no | yes | 12 Years and older (Child, Adult, Older Adult) - Healthy | yes | yes |
| NCT04816643/  2020-005442-42 | March 25, 2021 | June 1, 2021 | Study to Evaluate the Safety, Tolerability, and Immunogenicity of an RNA Vaccine Candidate Against COVID-19 in Healthy Children <12 Years of Age | Recruiting | Phase I /Phase II | no | no | no | no | no | yes | yes | yes | 6 Months to 11 Years (Child)  - Healthy | yes | yes |
| NCT04863638 | April 28, 2021 | May 13, 2021 | A Immuno-bridging and Immunization Schedules Study of COVID-19 Vaccine (Vero Cell), Inactivated | Recruiting | Phase IV | yes | no | no | no | no | yes | no | yes | 3 Years and older (Child, Adult, Older Adult) - Healthy | no | no |
| 2020-005720-11 | April 22, 2021 | - | A Randomized, Double-blind, Placebo-controlled, Phase 2/3 Study to Evaluate the Safety, Reactogenicity, and Immunogenicity of Different Dose Levels of Ad26.COV2.S Administered as a Two-dose Regimen Followed by a Booster in Healthy Children From Birth to 17 Years Inclusive When Compared to the Administration of One- and Two-doses of Ad26.COV2.S (in a Two-dose Regimen) in Healthy Adults Aged 18 to 55 Years Inclusive | Only outside EU (not started in EU). Only registered in EudraCT because of PIP) | Phase II /Phase III | yes | yes | yes | yes | no | yes | no | yes | from birth to 17 years of age  - Healthy | yes | yes |
| NCT04800133 | March 16, 2021 | April 12, 2021 | Covid-19 Vaccination in Adolescents | Recruiting | Phase II | no | no | yes | no | yes | yes | yes | yes | 11 Years to 100 Years  (Child, Adult, Older Adult) - Healthy | yes | no |
| NCT04916886 | June 8, 2021 | June 8, 2021 | A Clinical Trial of Immunobridging and Lot-to-lot Consistency of COVID-19 Vaccine (Ad5-nCoV) in Different Age Groups. | Recruiting | Phase III | yes | yes | yes* | no | no* | yes | no | yes | 6 Years to 59 Years (Child, Adult) - Healthy | no | no |
| NCT04917523 | June 8, 2021 | June 8, 2021 | Immuno-bridging Study of Inactivated SARS-CoV-2 Vaccine in Healthy Population Aged 3-17 vs Aged 18 Years Old and Above | Not yet recruiting | Phase III | no | no | no | no | no | yes | no | yes | 3 Years and older (Child, Adult, Older Adult) - Healthy | no | no |
| NCT04918797 | June 9, 2021 | June 9, 2021 | COVAXIN in a Pediatric Cohort | Recruiting | Phase II /Phase III | no | no | no | no | no | yes | no | yes | 2 Years to 18 Years  (Child, Adult) - Healthy | no | no |
| NCT04895982 | May 21, 2021 | May 21, 2021 | Study to Evaluate Safety, Tolerability & Immunogenicity of BNT162b2 in Immunocompromised Participants ≥2 Years | Not yet recruiting | Phase II | no | no | no | no | no | yes | no | yes | 2 Years and older (Child, Adult, Older Adult)  - Immunosuppressed | yes | no |
| 2021-000673-83 | Feb 19, 2021 | - | Impact of the immune system on response to COVID-19 vaccine in allogeneic stem cell recipients | Ongoing BE | Phase IV | no | no | no | no | no | yes | yes | yes | 12 years or older  - Immunosuppressed | yes | no |

*lot-to-lot comparison
